# Conference Report: APPLICATION PORTABILITY PROFILE AND OPEN SYSTEM ENVIRONMENT USER’S FORUM Gaithersburg, MD May 9–10, 1995

**DOI:** 10.6028/jres.100.052

**Published:** 1995

**Authors:** Joseph I. Hungate, Martha M. Gray, Kathleen A. Liburdy

**Affiliations:** Systems and Software Technology Division, National Institute of Standards and Technology, Gaithersburg, MD 20899-0001; Clemson University, Clemson, South Carolina 29634

## 1. Introduction

The National Institute of Standards and Technology (NIST), Systems and Software Division, sponsored a Users’ Forum on the Application Portability Profile (APP) and Open System Environment (OSE) at NIST in May. This forum was the fifteenth in a continuing semiannual series on the NIST APP and its application to OSE. The APP Users’ Forums are designed to provide users and providers with the opportunity to exchange information and respond to NIST proposals regarding the evaluation and adoption of an integrated set of standards to support the APP and OSE.

The forum offered the customary presentation of standards and activities in the APP, OSE, Institute of Electrical and Electronic Engineers (IEEE), and Joint Technical Committee 1 (JTC1-international activities). A workshop on Automated Testing Technologies was featured on the second day with extensive discussions concerning participants’ case studies, current activities, plans and lessons learned. A tutorial for beginners with little or no experience with the APP and OSE was held on the morning of the first day. The tutorial presented basic OSE concepts and the reference model.

The next APP/OSE Users’ Forums will be held May 7 and 8, 1996 at NIST.

The APP/OSE Users’ Forum has been developed to assist federal agencies with information technology (IT) issues. Central to this assistance is publication and maintenance of a technical guidance document, the Application Portability Profile (APP), facilitating the migration to open systems. An Open System Environment encompasses the functionality needed to provide interoperability, portability, and scalability of computerized applications across networks of heterogeneous, multi-vendor hardware/software/communications platforms. The APP integrates industry, federal, national, international, and other specifications into a Federal application profile to provide the functionality necessary to accommodate a broad range of Federal information technology requirements. The Application Portability Profile (APP), The U.S. Government’s Open System Environment Profile OSE/1 Version 3.0 provides recommendations on a variety of specifications that will generally fit the requirements of U.S. Government systems. A specific organization will not necessarily require all of the recommended specifications in the APP. As the U.S. Government’s OSE profile, this guidance is provided to assist Federal agencies in making informed choices regarding the selection and use of OSE specifications, and in the development of more selective application profiles based on the APP. It is directed toward managers and project leaders who have the responsibilities of acquiring, developing, and maintaining information systems supported by heterogeneous application platform environments.

## 2. Standards Status

Fritz Schulz, NIST, presented the following updates on the OSE standards activities of IEEE, JTC1 and the Computer Systems Laboratory (CSL) of NIST.

The IEEE Portable Application Steering Committee (PASC), which sponsors the Portable Operating System Interface (POSIX) projects has reorganized. Previously each standard activity had been individually numbered, and now activities are grouped into seven areas. These areas are system services, shells and utilities, system administration, language bindings, security, profiles, and test methods. In addition to lowering overhead and increasing efficiency, this reorganization will make it easier to progress approved standards to the international arena.

The OSE guide developed by P1003.0 has been approved and will be published very soon as a technical guide. The POSIX OSE guide describes an OSE Reference Model (OSE/RM) that is closely aligned with the APP and that provides a framework for describing open system concepts and defining a lexicon of terms that can be agreed upon generally by all interested parties. The same document is in ballot as a draft technical report (DTR) 14252 within working group (WG)15 of subcommittee (SC)22 of JTC1. The DTR is also expected to be approved very soon. The status of individual programs within the POSIX project were distributed in a handout.

Technical Report 10000-3: “Information Technology—Framework and Taxonomy of International Standardized Profiles—Part 3: Principles and Taxonomy for Open System Environment Profiles,” produced by the JTC1 Special Group on Functional Standardization (SGFS) has been approved and will be published very soon. TR 10000, part 3 provides a context for functional standardization in support of Open System Environments (OSE). It outlines the basic OSE objectives and concepts, and defines an approach to the taxonomy and format for OSE Profiles specified by International Standardized Profiles. The technical report gives guidance on the nature and content of International Standardized Profiles (ISPs) documents to organizations proposing Draft OSE ISPs.

### 2.1 Application Portability Profile (APP) Version 3

Gary Fisher, NIST, made the presentation on the new version of the APP.

A selected suite of specifications that defines the interfaces, services, protocols, and data formats for a particular class or domain of applications is called a profile. The Application Portability Profile (APP) integrates industry, Federal, national, international, and other specifications into a Federal application profile to provide the functionality necessary to accommodate a broad range of Federal information technology requirements.

The APP is *not* a standard and is not designed to cover every case. In some instances, the selection of one specification recommended in the APP will obviate the need for other specifications that are also recommended (i.e., select one or the other, but not both.) There is some overlap in functionality covered in different specifications. There are also gaps in functionality. In areas where the APP does not meet all of a user’s requirements, the user must augment the recommended specifications to ensure that proposed systems built on these specifications meet organizational requirements. The APP is designed to help users determine which specifications to use.

Not only is the U.S. Government involved in the development of profiles, but industry, national, and international organizations are preparing specifications that encompass numerous types of profiles. Corporations such as American Airlines, Boeing, DuPont, General Electric, Kodak, McDonnell Douglas, Merck, Motorola, Northrop, and Unilever are developing profiles for use within their own organizations and in many cases have based these profiles on the APP. The Institute of Electrical and Electronics Engineers, the International Organization for Standardization, and other standards-making organizations are in the process of developing profiles for specific types of application domains. U.S. Government organizations that are engaging the concepts of organizational profiles include the U.S. Army Sustaining Base Information Services, the U.S. Bureau of the Census, the Internal Revenue Service, the Defense Information Systems Agency, and many others.

Many specifications were reviewed and evaluated before the final recommended specifications were selected. If there are other specifications that should be considered in the APP and that meet a broad range of U.S. Government application requirements, users, vendors, and other interested parties should formally recommend them for evaluation using the same evaluation criteria applied to the selected specifications. This is one of the ways in which the APP will continue to evolve as technology evolves.

The initial version of the APP was published by the National Institute of Standards and Technology (NIST) in April 1991 as Special Publication 500-187. Version 2 of the APP Guide, NIST Special Publication 500-210, was published in June 1993. The changes in this third revision reflect the evolutionary developments that have occurred in the standards arena. Examples of the types of changes in this version include the following:

- The introductory material incorporates work done by the Institute of Electrical and Electronics Engineers (IEEE) POSIX Working Group 1003.0 on the Open System Environment Reference Model (OSE/RM).
- The evaluation criterion, *de facto usage*, has been removed and others have been reworded to provide more usable definitions.
- A new *bindings* information item has been added to individual specifications where appropriate.
- All of the recommended specifications have been updated and many new ones have been added. Areas that have seen the most change are those that encompass data interchange and communications where numerous new specifications have been added.

Specific changes between Version 2 and Version 3 recommended specifications include the following:

- Operating System Services
  IEEE 1003.2-1992 POSIX Shells and Utilities is now FIPS 189.
  IEEE 1003.4 Realtime is now IEEE 1003.1b.
  IEEE 1003.6 Security is now IEEE 1003.1e and IEEE 1003.2c.
  IEEE P1387.2, .3, and .4 are new.
- Human/computer Interface Services
  Proposed FIPS 158-1 X Window System is now officially FIPS 158-1.
  IEEE P1295 X Window Toolkit is now IEEE 1295.1.
- Software Engineering Services
  FIPS 119 Ada is now FIPS 119-1 Ada.
  FIPS 21-3 COBOL is now FIPS 21-4 COBOL.
  FIPS 119 Pascal has been deleted due to very limited interest in this specification.
  ECMA PCTE has been replaced by ISEE Repository ISO/IEC 13719-1.
- Data Management Services
  FIPS 127-1 SQL is now FIPS 127-2.
  FIPS 193 SQL Environments is new.
- Data Interchange Services
  ODA/ODIF/ODL ISO 8613 has been deleted due to lack of implementations.
  Draft Portable Document Delivery Format (PDDF) is new.
  SPDL ISO 10180 has been deleted and replaced by PDDF.
  Standard Data Elements ISO 11179 Parts 3, 4, and 5 are new.
  FIPS 194 Raster is new.
  JPEG is new.
  MPEG is new.
  STEP ISO 10303 has been replaced by the planned FIPS on STEP.
  FIPS 173 SDTS is now FIPS 173-1.
- Graphics Services
  FIPS 153 PHIGS is now FIPS 153-1.
- Network Services
  PII API P1003.12 has been renamed P1003.1g.
  IEEE 1238.1 FTAM has been deleted. (This specification is part of FIPS 146-2.)
  FIPS 146-1 GOSIP is now FIPS 146-2 POSIT.
  ISDN is now FIPS 182 ISDN.
  IEEE 1003.8 TFA has been deleted. (This specification is part of FIPS 146-2.)
  CORBA is new.
  FIPS 179 GNMP has been deleted and replaced with OMNIPoint.
  FIPS 192 GILS is new.
  NISO Z39.50 is new.
  FIPS 46-2 DES is new.
  FIPS 186 DSS is new.

The universe of OSE is continually evolving and the APP Guide will strive to reflect this evolution. The Computer Systems Laboratory (CSL) welcomes any recommendations for changes to the APP.

### 2.2 Profiles for Open System Internetworking Technology (POSIT)

Tassos Nakassis, NIST, reported that the Secretary of Commerce recently approved two revised standards: FIPS 146-2, Profiles for Open Systems Internetworking Technologies (POSIT), and FIPS 179-1, Government Network Management Profile (GNMP). Effective immediately, FIPS 146-2 removes the requirement that federal agencies specify Government Open Systems Interconnection Profile (GOSIP) protocols when they acquire networking products and services and communications systems and services. FIPS 179-1 provides implementations for network management based on the service and protocol standards issued by the International Organization for Standardization (ISO). These revised standards promote the interoperability of computers and systems that are acquired from different manufacturers in an open systems environment.

### 2.3 Document Management Services

Mike Rubinfeld, NIST presented the status of three standards used in document management, Joint Photographic Experts Group (JPEG), Moving Pictures Experts Group (MPEG) and Portable Document Delivery Format (PDDF). JPEG is being developed under the auspices of ISO/IEC JTC1/SC2 Working Group 10. The current standard, IS 10918:1992, specifies the digital compression and coding of continuous-tone still images. These images can be either grayscale or color. The standard uses 24 bit compression and consists of three elements, an encoder, a decoder, and the interchange format. ISO 10918:1992 uses other standards as well. They are SGML Z39.50, MPEG, Huffman Encoding and ISO/IEC IS9660.

ISO/IEC JTC1/SC2 Working Group 11 is the sponsor of the IS 11172:MPEG-1 standard. The standard is for video compression for multimedia applications. It addresses compression of video signals up to 1.5 Mbits/s. MPEG audio compresses the audio signal at rates of 64, 128 and 192 kbits/s. MPEG is used in conjunction with mass media such as hard drives, CD-ROM and other optical storage, writable CD, DAT tape, and network servers.

MPEG utilizes two techniques, blocked-based motion compression—reduction of temporal redundancy and transform domain-based compression—reduction of spacial redundancy (DCT).

PDDF is based on a blue ribbon panel’s recommendations and a set of basic requirements for a standard. Final Form Portable Document Delivery Format consists of encoded representation on electronic medium in presentation quality final form. The current situation requires the use of proprietary formats resulting in conversion nightmares that often require resorting to ASCII as a common denominator. The PDDF project goals are:

- Identify Needs within the Government
- Develop a set of Requirements
- Assess the Current Technology
- Describe a PDDF that meets the Requirements
- Develop a Conformance Test Suite Based on the PDDF
- Draft a FIPS for the Preferred PDDF

To meet these goals, government user and vendor workshops were held with NIST serving as an overall catalyst, coordinator and initiator of cooperative research and development agreements (CRADAs) with vendors. NIST will also provide documents from workshops, develop a conformance test plan and consider PDDF as a future FIPS.

PDDF provides new way to preserve documents that will alleviate costs associated with conversion and use of unnecessary software. This will make the use of electronic medium for document exchange much easier. Storage cost and paper cost savings will be significant.

The baseline set of requirements for choosing a format was developed in the Open System Environment Implementors Workshop (OIW) from contributions by vendors and the Blue Ribbon Panel. A set of 19 requirements was established to serve as a guide for selecting a PDDF. The project will also address the following recommendations from the Blue Ribbon Panel:

- Conformance Verification—Provide for software conformance to the format specification via a conformance test plan and associated test suite. Provide a registry of conformant software products.
- Organize a Users PDDF Forum comprised of government users and industry developers.

### 2.4 SQL Standards and FIPS 127-2

Joan Sullivan, NIST, gave the presentation on SQL and the associated FIPS. First introduced in 1986, FIPS 127 (SQL-86) addressed only basic functionality. In 1989, integrity enhancement was added resulting in FIPS 127-1 (SQL-89). 1992 saw the issuance of FIPS 127-2 (SQL-92), with a four level structure. The levels are entry, transitional, intermediate, and full. The next revision of FIPS 127-2 will be based on SQL-9x, which will consist of six major parts.

The SQL conformance testing began in 1988 with 191 tests growing to 384 by December of 1989. In April of 1990, NIST started a SQL trial testing service, and issued registered validation summary reports. The trial period ended in 1992, and testing certificates started being issued in 1993. Currently tests exists for FIPS 127-1 and two levels (entry and transitional) of FIPS 127-2. Additional levels of FIPS 127-2 will be available in 1996. The FIPS 127-1 validated product list contains 12 companies, offering 14 products. The list for FIPS 127-2 has six companies, offering 12 products. There is worldwide interest in SQL testing. The NIST test suite is licensed internationally in Australia, Belgium, Canada, China, France, Germany, Italy, Greece, Japan, Korea, Sweden, United Kingdom, and the USA.

To ensure portability of SQL programs, a simple FIPS 127-2 strategy is necessary. Users should specify FIPS 127-2 conformance in request for proposals (RFPs) and require a test certificate. On existing database products users should upgrade to validated products. Most importantly, they should educate development staff on standard SQL and enforce its use in application development.

### 2.5 Digital Encryption Standard (DES) and Digital Signature Standard (DSS)

Lisa Carnahan, NIST, presented the status of FIPS 46-2, DES, and FIPS 186, DSS. FIPS 46 was first issued in 1977 to protect unclassified information from unauthorized disclosure or modification. NIST reviews the standard every 5 years, and has reaffirmed it at its last review in 1993. As a result of that review, use of software implementations is now allowed in addition to hardware implementations. DES is documented and is validated in accordance with NIST SP 500-20. The validation test entails using a NIST supplied key and 64 bit input and then performing 8 million encryptions and 4 million decryptions.

FIPS 186, DSS, was issued in May of 1994. The standard contains an algorithm to use in designing and implementing public-key based signature systems. A companion FIPS 180-1 for a secure hash standard (SHS) was issued in April of 1995 for use when computing a condensed representation of a message or data file. Any change in the message will, with a high degree of probability, result in a different result. DSS conformance tests are modular, consisting of signature generation, signature verification, primality tests, global parameter generation (p,q,g), key generation (x,y), and per message parameter generation (k). All implementations must generate k (per message parameter) and sign or verify.

### 2.6 Standard for the Exchange of Product Model Data

A FIPS has been proposed for the Standard for the Exchange of Product Model Data (STEP) that will adopt the voluntary industry specification International Organization for Standardization (ISO) Product Data Representation and Exchange, ISO 10303:1994. STEP defines and describes all product data used during the manufacturing life-cycle of a product, the production steps needed to make and product, and the order in which they occur. Comments on this proposed standard are welcomed. The proposed FIPS is available from the CSL Office.

### 2.7 Standard Generalized Markup Language (SGML)

Ron Wilson, NIST reported on a task initiated by the CALS Project Office to organize an SGML Conformance Testing Service. The NIST SGML Conformance Testing Program will certify that SGML parsers meet the requirements of the Federal Information Processing Standard (FIPS) 152. The Computer Systems Laboratory of the National Institute of Standards and Technology (NIST) is responsible for establishing conformance testing programs for Federal Information Processing Standards (FIPS). In carrying out this responsibility, CSL specifies the necessary conformance test specifications, test methods (i.e., test suites, test tools, and technical procedures), validation procedures, and testing laboratories for testing product compliance to FIPS.

NISTIR 5538, SGML Parser Validation Procedures, establishes operating policy and procedures for the Computer Systems Laboratory’s (CSL) validation program for Federal Information Processing Standards (FIPS) 152, Standard Generalized Markup Language (SGML) parsers. The testing methodology is based on ANSI X3.190-1992, Text and Office Systems—Conformance Testing for Standard Generalized Markup Language Systems. This document contains operating policy for a Standard Generalized Markup Language Conformance Testing Service and is not intended to explain the detailed procedures that can be found in the documentation associated with the SGML parser validation system, commonly called the SGML Test Suite.

### 2.8 POSIX.2 Shells and Utilities

Sheila Frankel, NIST Portable Operating System Interface (POSIX)—Part 2: Shells and Utilities provides a command language interpreter (shell) and a set of utility programs that promotes user and application portability. It is used for directory/file/data creation and manipulation, interaction with the operating system, and automation of repetitive tasks. POSIX part 2, shells and utilities is the subject of FIPS 189, and is based on ISO/IEC standard 9945-2:1993. This standard is also known as ANSI/IEEE Standard 1003.2-1993 or POSIX.2. The effective date of FIPS 189 is April 3, 1995. FIPS 189 is required for operating systems and/or applications development where POSIX shell and utility interfaces are required. FIPS 189 adopts the POSIX.2 Standard, but omits obsolescent features, or violation of the general syntactic guidelines of POSIX.2 that may be deleted from POSIX.2 at a future date. POSIX.2 requires that these features not be used by strictly conforming applications. Most obsolescent features have equivalent, nonobsolescent counterparts in POSIX.2. A FIPS 189 Testing Program is being developed by NIST. It will be similar to the conformance testing program that was established for FIPS 151-2. The testing program will use accredited Labs from the National Voluntary Laboratory Accreditation Program (NVLAP) to perform the testing. Certificates of Validation will be issued by NIST and an accredited products list maintained. The FIPS 189 Conformance Testing Program will adopt an existing test suite. A call for available test technology was published in the Commerce Business Daily (CBD) was published on October 20, 1994. Test suites submitted in answer to that announcement have been evaluated and an in-house report has been issued. An advisory board has been convened that will publish the testing model, test suite criteria, and select test suite(s). A follow-on activity, POSIX 2003.2 is under way. Upon approval the Test Methods for POSIX.2 standard will result in a test suite(s) update.

For further information contact Sheila Frankel at (301) 975-3297 or frankel@sst.ncsl.nist.gov.

### 2.9 Open System Environment Implementors’ Workshop (OIW)

Joe Hungate, OIW chairman, made the presentation on the OIW. The Open Systems Environment Implementors Workshop is a public international technical forum for the timely development of implementation agreements based on emerging international standards and public specifications. Its purpose is to broaden the utilization of Open Systems Environment (OSE)-based technologies and to speed their development. The workshop intent is to support the advancement of a technically efficient and compatible technology base for emerging Open Systems on a nationwide basis. NIST chairs the OIW, which meets quarterly at NIST. Workshop organizational components include the Plenary (now conducted electronically), two standing committees—the Technical Liaison Committee (TLC) and the Open System Environment Technical Committee (OSETC), and Special Interest Groups (SIGs) that perform the technical work. The Plenary reviews and ratifies SIGs technical programs of work. SIGs may also have subsidiary working and study groups to address specific issues. The workshop also consists of various Working Groups and Special Project Teams created to deal with emerging technologies and issues.

For additional information on the OIW, please contact Joe Hungate at (301) 975-3368, or jhungate@nist.gov.

## 3. Automated Testing Technologies Workshop

The workshop presented with the assistance of Clemson University, hosted a second workshop on automated testing technologies. The goals of this workshop, like the first, included reviewing existing and emerging technologies for automated specification and development of test methods, exploring the relationship between automated testing and standards development, establishing a forum for the continuing exchange of information between experts working in this area, and proposing an agenda for action which will support and accelerate efforts in automated testing.

The three focus areas of this workshop were the ADL Project, presentations on university experiences and formal methods, and industry experiences.

### 3.1 The ADL Project

#### 3.1.1 Update on the Assertions Definition Language (ADL) Project

Shane McCarron, Testing Research Manager, X/Open Company Ltd., presented an overview of the Assertion Definition Language Project. This overview provided a brief history of the project, described in some detail the activities over the last year, and presented a high level view of the activities planned for the coming year. The presentation also included a description of last year’s efforts to use ADL to generate a test suite for the CORBA (Common Object Request Broker Architecture specification) 1.2 specification and a test suite for TET (a public domain, joint industry developed test harness).

McCarron described ADL as “a (semi) formal language in which it is possible to describe the behavior of interfaces. The ADLTranslation System is a collection of tools and additional ‘languages’ that permit the specification and generation of natural language interface specifications, test specifications, and tests based upon the ADL interface specification.” The goals of ADL are to improve test coverage, reduce costs of test development, speed up the process of test suite generation, reduce lifecycle costs and improve the reliability of testing.

#### 3.1.2 ADLT in Practice: Experiences and Anecdotes

Roger Hayes of Sun Microsystems Laboratories described the ADLT as a freely-available system for test software generation, developed by Sun Laboratories with the support of X/Open and Japan’s Ministry of International Trade and Industry (MITI/IPA). Hayes described, briefly, some of the large-scale testing projects that have adopted ADLT, i.e., OpenDoc, OpenGL, PIKS, OMG CORBA, and TET, and lessons that have been learned in the course of supporting those projects. The lessons learned included that ADLT works, that there is a high degree of re-use, that it is useful in clarifying specifications, and that it can be used to develop portable tests.

### 3.2. University Experiences and Formal Methods

#### 3.2.1 Exploration of Easy-to-Use Formalisms for Software Specification

The project results of a 1 year collaborative effort between Sun Microsystems Laboratories and Clemson University were reported in this presentation by Kathy Liburdy of Clemson University. The goal of this effort was to provide insights on the ease of learning and using a software specification language such as the Assertion Definition Language (ADL) developed by Sun Microsystems Laboratories.

A classroom experiment involving senior students in Clemson University’s Computer Engineering Program was designed to provide both a unique learning experience for the students as well as to achieve the desired goals of the collaborative effort.

The classroom experiment consisted of two distinct phases: tutorial development and specification development. Phase one required the students to develop a tutorial for ADL based on the ADL Language Reference Manual. This effort served the dual purpose of familiarizing the students with ADL and identifying difficulties with the Language Reference Manual. There were three teams involved in the experiment, and three very different tutorials resulted. An overview of the tutorials as well as observations regarding the Language Reference Manual were reported.

The second phase of the experiment required the students to develop specifications using ADL for a relatively simple problem such as a symbol table manager. After the specifications were developed, the students exchanged specifications and were asked to implement software satisfying another team’s specification. As the final component of the experiment, implementations were returned to the specification developers for evaluation. Students’ comments on the ease of learning ADL, using ADL, and implementing software from ADL specifications based on this experience were reported. Suggestions for supplemental material for teaching ADL concluded the presentation.

#### 3.2.2 Automated Test Methods for POSIX.5

This presentation by Jim Leathrum of Clemson University, described the development and transfer of an automated testing technology in the open systems standards arena which resulted from a government and university alliance. The Clemson Automated Testing System (CATS) was initiated in response to the U. S. Navy’s request for conformance testing technology. The vision during the effort was a system which would provide life cycle support for conformance testing (i.e., assertion writing, test generation, test execution, and test results analysis). The system design was based upon the traditional compiler paradigm in that assertions about the required behavior of an implementation under test are translated into executable tests.

In 1994, the Defense Information Systems Agency (DISA) sponsored the application of the CATS technology in the development of test methods for the Ada Language binding to POSIX. The development of assertions for POSIX.5 revealed significant realizations which can be directly attributed to the technology, such as the value of providing rapid feedback from the CATS environment during test development. Additionally, Leathrum disclosed that it became apparent that testing issues were addressed much earlier in the process than in traditional approaches. A discussion of lessons learned from this experience concluded the presentation.

#### 3.2.3 Symbolic Execution and Constraint Satisfaction in Automatic Test Case Generation

Steven Zeil from Old Dominion University submitted a paper which described the following.

For over 20 years, researchers have noted that symbolic execution offered a conceptually elegant approach to the automatic generation of tests for structural and other implementation-based testing criteria.

A number of symbolic execution systems have been built, typically for older programming languages and offering limited facilities for constraint satisfaction. The inability of these systems to deal with abstract data and more modern programming languages has raised questions about the viability of symbolic execution in general.

The ARIES symbolic executor attempted to modernize symbolic execution, allowing symbolic execution of Ada programs. Although its runtime performance was disappointing, it offered some significant advances in design, including:

- Preservation of abstraction in expressions involving ADT’s
- Separation of constraint satisfaction from the execution engine

Advances in constraint satisfaction techniques also hold new promise. Test case generation has unusual characteristics that place a strain on constraint solvers. The constraint systems seldom fall into the neat classifications on which most solvers are designed, but tend to mix many constraint theories. On the other hand, some recent projects have indicated that the vast majority of constraint systems that arise during testing tend to be easily solvable, despite being ill-formed for conventional techniques. Some preliminary experience with a MTCSS (Multi-Theory Constraint Satisfaction System) suggests directions for future work.

### 3.3. Industry Experiences

#### 3.3.1 Automatic Efficient Test Generator (AETG) System

According to David Cohen, Bellcore, software testing is expensive, tedious and time consuming. By some estimates, testing accounts for 30 % to 50 % of development costs. Making testing more efficient has long been a goal of software engineering research. Cohen described the Automatic Efficient Test Generator (AETG) System that is a new tool developed by Bellcore that automatically generates test sets from high-level test requirements. It uses new algorithms from combinatorial design theory to generate test sets that efficiently cover the test requirements. The AETG system has been used in Bellcore and a major telecommunications manufacturer for feature and protocol conformance testing, for inter-operability testing, and for testing user interface software. Cohen presented data from these experiences.

#### 3.3.2 TGGS: A Flexible System for Generating Efficient Test Case Generators

Ronald F. Guilmette, RG Consulting, described the Test Generator Generator System, TGGS. TGGS is a simple yet flexible system for generating highly efficient automated random test case generators. The random test case generator programs generated by TGGS may themselves used to generate randomized test cases for a variety of programs, most notably compilers. TGGS is based upon a specification language (SL), very similar to the input language accepted by YACC, in which the user may express both the syntactic and semantic constraints of the input language for the program to be tested. The SL language was described in detail. A description of the SL compiler, GTG, and of the associated SL runtime system was also provided, and the application of TGGS to some example testing problems described.

#### 3.3.3 A Solution for Automated Testing to Ensure Product Interoperability

John Reardon from Midnight Networks Inc. described his views on testing networking products. He said that thorough testing is a requirement for network products to interoperate acceptably in customer networks. However, it is not possible to perform such testing solely via manual methods and test net operation, as such approaches are costly, slow and ineffective.

Automated testing allows thorough, rigorous testing to be performed, while cutting the time it requires. By using automated testing with positive conformance tests, negative tests, and stress tests, interoperability may be achieved. Reardon described the design and architecture of a system for automated network testing that has been implemented by Midnight Networks. He also described experiences and case histories that show its benefits in cost, cycle time and quality to organizations that make use of it.

#### 3.3.4 Automated Test Generation with TestMaster

The presentation by Larry Apfelbaum, Teradyne Software & Systems Test, described a new approach that has been successful for automating the test generation phase for software based systems. This solution uses a model of an application’s desired behavior as the basis for a flexible, automated test generator. The presentation covered the process of modeling and how a path generation engine operating with that model can efficiently generate tests of a known quality. Included were a description of the elements of a model and the role they play as the basis of a testable specification. The process a path generator uses to build tests was also explained with some examples. Samples of the results obtained by some of the existing production applications of this technology were given.

#### 3.3.5 STEP Conformance Testing

The NIST (National Institute of Standards and Technology) and ITI (Industrial Technology Institute) program on STEP Conformance Testing is entering its fourth year. Bob Matthews from ITI presented a review and update of the status of this program. To date, this program has developed a variety of tools, tests, and services enabling STEP product conformance testing. The testing technology is currently being used by many vendors, users, test laboratories, and standards developers, and is accelerating STEP product realization.

The goals of the fourth year of the program are to extend the features of several prototypes, complete integration of tools into a CASE-style environment, launch a prototype conformance test service, apply conformance test technology to support interoperability testing, adapt tools and services to enable testing of related standards, and validate test technology and methods.

Several tools which are being extended were described including the: Coverage Analyzer (CA), ARM/AIM Browser/Editor (AABE), and Verdict Criteria Generator (VCGEN). The CA serves two principal purposes: verification of conformance test suite completeness and quantitative analysis of interoperability tests. The AABE tool provides application-domain views of STEP exchange data. The VCGEN tool produces sets of detailed verdict criteria that are used to efficiently evaluate testing outputs.

Matthews described the STEP Test System (STS) which integrates the complete set of STEP conformance test tools. The STS provides an object-oriented testing paradigm to enable users to quickly learn and manipulate the many artifacts involved in conducting tests. The core of the STS is a testing “harness” which enables simplified plug-in capability for standard-specific test tools and data.

Matthews also described a “beta” STEP conformance testing service which was recently launched. Using the conformance test technology developed under the NIST-ITI program, early STEP vendors engage the service to formally evaluate and demonstrate to users the conformance, and therefore interoperability potential, of their products. This beta service is expected to evolve into a replicable NIST-NVLAP accredited STEP testing process.

Matthews summarized that to date, most of the testing program efforts have focused on working with one principal STEP standard, AP 203. Dozens of other STEP standards will be coming on-line, and require similar testing support. The NIST-ITI tools, designed to serve the many standards of STEP, are now being configured, extended, and applied to these other STEP standards.

## 4. Tutorial for Novices

The forum began with an introductory tutorial on the Open System Environment (OSE). The OSE forms an extensible framework that allows services, interfaces, protocols, and supporting data formats to be defined in terms of nonproprietary specifications that evolve through open (public), consensus-based forums. A selected suite of specifications that defines these interfaces, services, protocols, and data formats for a particular class or domain of applications is called a profile. Fritz Schulz presented OSE general concepts and the reference model.

### 4.1 Open System Environment (OSE) Reference Model (RM)

**Fig. 1:**
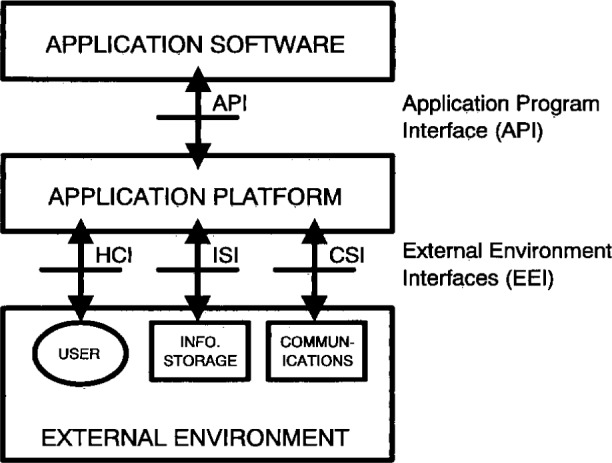
Open System Environment Reference Model (OSE/RM).

The Institute of Electrical and Electronics Engineers (IEEE) POSIX Working Group 1003.0 defines an OSE Reference Model (OSE/RM) that provides a framework for describing open system concepts and defining a lexicon of terms that can be agreed upon generally by all interested parties. The OSE/RM is also identified at the international level in Joint Technical Committee 1 (JTC1) Technical Report (TR) 14250. Figure 1 illustrates the OSE/RM.

Two types of elements are used in the model: entities consisting of the application software, application platform, and platform external environment; and interfaces including the application program interface and external environment interface.

The three classes of OSE reference model entities are described as follows:

- *Application Software*—Within the context of the OSE Reference Model, the application software includes data, documentation, and training, as well as programs.
- *Application Platform*—The application platform is composed of the collection of hardware and software components that provide the generic application and system services.
- *Platform External Environment*—The platform external environment consists of those system elements that are external to the application software and the application platform (e.g., services provided by other platforms or peripheral devices).

There are two classes of interfaces in the OSE reference model: the application program interface and the external environment interface.

- *Application Program Interface (API)*—The API is the interface between the application software and the application platform. Its primary function is to support portability of application software. An API is categorized in accordance with the types of service accessible via that API. There are four types of API services in the OSE/RM:
  1. Human/computer interface services
  2. Information interchange services
  3. Communication services
  4. Internal system services
- *External Environment Interface (EEI)*—The EEI is the interface that supports information transfer between the application platform and the external environment, and between applications executing on the same platform. Consisting chiefly of protocols and supporting data formats, the EEI supports interoperability to a large extent. An EEI is categorized in accordance with the type of information transfer services provided. There are three types of information transfer services. These are transfer services to and from:
  1. Human users
  2. External data stores
  3. Other application platforms

In its simplest form, the OSE/RM illustrates a straightforward user-supplier relationship: the application software is the user of services and the application platform/external environment entities are the suppliers. The API and EEI define the services that are provided.

### 4.2 OSE Profile and the APP

A profile consists of a selected list of standards and other specifications that define a complement of services made available to applications in a specific domain. Examples of domains might include a workstation environment, an embedded process control environment, a distributed environment, a transaction processing environment, or an office automation environment, to name a few. Each of these environments has a different cross-section of service requirements that can be specified independently from the others. Each service, however, is defined in a standard form across all environments.

An OSE profile is composed of a selected list of open (public), consensus-based standards and specifications that define services in the OSE/RM. Restricting a profile to a specific domain or group of domains that are of interest to an individual organization results in the definition of an organizational profile. The Application Portability Profile (APP) is an OSE profile designed for use by the U.S. Government. It covers a broad range of application software domains of interest to many Federal agencies, but it does not include every domain within the U.S. Government’s application inventory. The individual standards and specifications in the APP define data formats, interfaces, protocols, or a mix of these elements.

### 4.3 APP Service Areas

The services defined in the APP tend to fall into broad *service areas*. These service areas are:

- operating system services (OS)
- human/computer interface services (HCI)
- data management services (DM)
- data interchange services (DI)
- software engineering services (SWE)
- graphics services (GS)
- network services (NS)

**Fig. 2:**
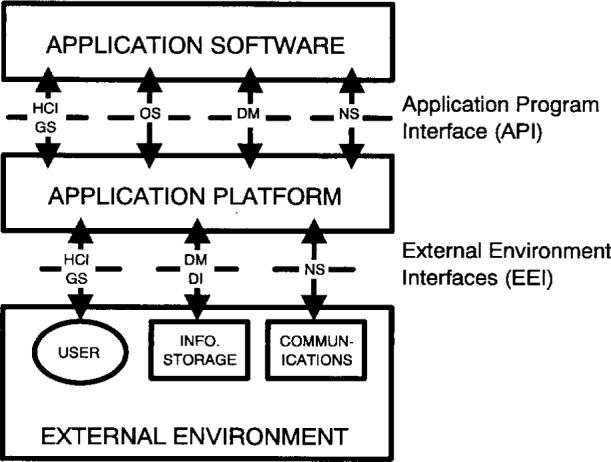
APP Service Areas and the OSE/RM.

Each service area is defined in the following sections. Figure 2 illustrates where each of these services areas relates to the OSE/RM. (Assume that software engineering services are applicable in all areas.)

Each of the APP service areas addresses specific components around which interface, data format, or protocol specifications have been or will be defined. Security and management services are common to all of the service areas and pervade these areas in one or more forms.

*Security* as applied to both stand-alone and distributed systems takes a holistic approach. Each component provides different elements of functionality and security service. Security services are provided to support the secure distribution and integrity of information and to protect the computing infrastructure from unauthorized access. Security policy, authority, domains, and interactions among these domains are specifically defined in IEEE P1003.22 *Draft Guide for POSIX Open Systems Environment—A Security Framework*. Security is a cross-category service and part of the overall context in which information systems must operate. It is of relevance within all system functions, for example system services, communications services, and data management services.

Currently, specifications for security can be recommended in operating system services, network services, and access control and integrity constraints for data management services. Specifications for security in the other service areas are not sufficiently advanced to warrant inclusion at this time.

*Distributed system management* is coming to be regarded as the integration of distinct, supporting management areas. Among these areas are system administration, communication (network) management, information management, and human/computer interface management. Management services provide the mechanisms to monitor and control the operation of individual applications, databases, systems, platforms, networks, and user interactions with these components. Management services also enable users and systems to become more efficient in performing required work.

These services are just now being addressed by standards development organizations (SDO) and user consortia, particularly for heterogeneous systems. The disparate mechanisms necessary for competent management of distributed systems require an integrated approach to assure consistency. Standardization is being developed by many committees in various SDOs, workshops, and consortia. Recent attempts by these committees has led to closer coordination. True integration among them, however, requires significant additional effort. As specifications for management services mature and stabilize, they will be reviewed and appropriate ones may be selected for use in the APP.

## 5. General References

Gary Fisher, Application Portability Profile (APP), The U.S. Government’s Open System Environment Profile OSE/1 Version 2.0, NIST Special Publication 500-210, June 1993.

